# ﻿Outline of the history and status of the Japanese Chrysomelidae (Coleoptera)

**DOI:** 10.3897/zookeys.1252.150709

**Published:** 2025-09-19

**Authors:** Kunio Suzuki, Haruo Takizawa

**Affiliations:** 1 13-45 Minami-Taikôyama, Imizu-shi, Toyama-ken, 939-0364 Japan Unaffiliated Minami-Taikôyama Japan; 2 Petit Monde Suwa Lake 1310, 9548 Kita-Takagi, Shimosuwa-machi, Suwa-gun, Nagano-ken, 393-0033 Japan Unaffiliated Shimosuwa-machi Japan

**Keywords:** systematics, zoogeography

## Abstract

The status of the Japanese fauna of the family Chrysomelidae is reviewed from the viewpoints of systematics and zoogeography based on a new systematic catalog in which 648 species belonging to 172 genera in 17 subfamilies were compiled by Takizawa and Suzuki. The rich Japanese chrysomelid fauna reflects a remarkable diversity of its natural environments. The number of species is relatively high in five subfamilies: Alticinae, Galerucinae, Eumolpinae, Chrysomelinae, and Cryptocephalinae. The percentage of indigenous species (275) compared with the total number of species exceeds 42.4%. The local faunas are compared among 10 regions. The history of studies on the Japanese Chrysomelidae is presented in three stages, Stage I (1758–1850), Stage II (1851–1950), and Stage III (1951–2023). Changes in number of species are presented for every 10 years period from 1758 to 2023. The cumulative number of species in the whole family clearly demonstrates a unique pattern with two remarkable increase times. We chose 10 representative coleopterists who contributed much to the elucidation of the Japanese chrysomelid fauna.

## ﻿Introduction

The family Chrysomelidae is one of the largest in the Coleoptera and it forms, along with two allied families Bruchidae and Cerambycidae, the huge superfamily Chrysomeloidea which is comprised of more than 63,000 described species (Ślipiński et al. 2011).

Since the mid-19^th^ century when two great pioneer workers, Chapuis (1874, 1875) and Jacoby (1908), laid the foundations of a basic higher classification system of the Chrysomelidae, a number of systems have been proposed by many workers. Suzuki (1996) reviewed the transition until 1995 in constructing a system of higher classification of the family above the subfamily level. After that, Löbl and Smetana (2010) published a comprehensive catalog of all the Palaearctic species. Recently Bezděk and Sekerka (2024) published a second revised version. Lawrence et al. (2011) compiled all the family-group names of Coleoptera of the world. For last several decades many phylogenetic works reflect various viewpoints including comparative morphology, comparative ecology, molecular biology, and so on. Concerning the superfamily Chrysomeloidea, especially the family Chrysomelidae s.l., the following works are important: Suzuki (1988), Kuschel and May (1990), Suzuki and Furth (1992), Suzuki (1994a, 1994b), Reid (1995, 2000), Kuschel and May (1996), Furth and Suzuki (1998), Suzuki and Windsor (1999), Suzuki (2003), Gómez-Zurita (2004), Gómez-Zurita et al. (2005), Hunt et al. (2007), Gómes-Zurita et al. (2007, 2008), Jolivet and Verma (2008), Bouchard et al. (2011), Jolivet et al. (2013), Haddad and McKenna (2016), Nie et al. (2017, 2018, 2019, 2020), Douglas et al. (2022), Zhang et al. (2022). There are differences among these works, but we will not discuss the systematic and phylogenetic relationships among higher taxa here. In our opinion, no reliable system of higher classification system has yet been established.

Recently we edited a new systematic catalog (“New Catalog”) of the families Bruchidae and Chrysomelidae from Japan (Takizawa and Suzuki, in press). In the present paper we outline the history and status of the Japanese Chrysomel­idae based on our “New Catalog”. The higher classification system which we adopted in the catalog is almost established up to the end of the last century. However, many differences among recent systems exist in the change of taxon­omic ranking of certain subgroups of Chrysomelidae.

## ﻿Results and discussion

Table 1 shows a system of higher classification that we provisionally adopted in our “New Catalog” of the Japanese Chrysomelidae. We based our system mainly on comparative morphology of hindwing venation, male and female external and internal reproductive systems, and life history, biology, and ecology. Our classification of Chrysomelidae includes 20 subfamilies. Of the 20 subfamilies, three with asterisks (*), Palophaginae, Aulacoscelidinae and Megascelidinae, are not distri­buted in Japan. The subfamily Palophaginae is distributed in the Australian region (Australia; Kuschel and May 1990) and a part of the South America (Chile and Argentina; Kuschel and May 1996). The two subfamilies Aulacoscelidinae and Megascelidinae are distributed in the New World only (Seeno and Wilcox 1982).

**Table 1.** The higher classification system adopted in our “New Catalog” of the Japanese Chrysomelidae.

Superfamily	Chrysomeloidea
Family	Cerambycidae
Family	Bruchidae
Family	Chrysomelidae
Subfamily	Megalopodinae
Subfamily	Zeugophorinae
Subfamily	Palophaginae*
Subfamily	Aulacoscelidinae*
Subfamily	Orsodacninae
Subfamily	Synetinae
Subfamily	Sagrinae
Subfamily	Donaciinae
Subfamily	Criocerinae
Subfamily	Chrysomelinae
Subfamily	Galerucinae
Subfamily	Alticinae
Subfamily	Clytrinae
Subfamily	Cryptocephalinae
Subfamily	Lamprosomatinae
Subfamily	Chlamisinae
Subfamily	Hispinae
Subfamily	Cassidinae
Subfamily	Megascelidinae*
Subfamily	Eumolpinae

* These 3 subfamilies with * are not distributed in Japan.

Japan is about 378,000 km^2^ in the total land area. It consists of the four major islands (Hokkaido, Honshu, Shikoku, and Kyushu) and about 14,000 islands including the Nansei Islands [= Southwestern Islands]. The Nansei Islands is a generic name of multiple islands of various sizes. Japan is located at latitude 20.2–45.3°N and longitude 123–154°E ranging from the southernmost region of the Subarctic Zone (Hokkaido) to the northernmost region of the Subtropical Zone (Nansei Islands), and is completely surrounded by the Japan Sea, the East China Sea, and the Pacific Ocean. The rich Japanese chrysomelid fauna reflects a remarkable diversity of the natural environments.

Chûjô and Kimoto (1961) published the first comprehensive catalog of the Japanese Chrysomelidae. They followed the system of higher classification of that time in which the superfamily Chrysomeloidea consisted of three families Cerambycidae, Bruchidae, and Chrysomelidae, and the Japanese Chrysomelidae were classified into the following 16 subfamilies: Orsodacninae, Zeugophorinae, Megalopodinae, Donaciinae, Criocerinae, Cryptocephalinae, Lamprosominae (=Lamprosomatinae), Chlamisinae, Eumolpinae, Chrysomelinae, Synetinae, Galerucinae, Alticinae, Hispinae, and Cassidinae. Ohno (1971) and Kimoto (1994) accepted that system. Until a comprehensive catalogue of the Palaearctic Chrysomelidae edited by Löbl and Smetana (2010) was published, most of the Japanese coleopterists also accepted the system.

Suzuki (1988, 1994a, 1994b) proposed the following higher phylogenetic classification system in which 19 tribes belong to seven lineages mainly based on the results of comparative morphology of the internal reproductive systems of both sexes and hind wing venation: 1. Orsodacninae [(Lepturinae; Cerambycidae) + Orsodacnini], 2. Megalopodinae [(?Lamiinae; Cerambycidae) + Zeugophorini + Megalopodini], 3. Sagrinae [Sagrini + Donacini + Criocerini], 4. Synetinae [Synetini], 5. Chrysomelinae (Aulacoscelini + Chrysomelini)], 6. Galerucinae [Galerucini + Alticini], 7. Clytrinae [(Clytrini + Cryptocephalini) + (Chlamisini + Lamprosomatini) + (Hispini + Cassidini) + (Megascelini + Eumolpini)]. After that, the following subfamilies were added: Palophaginae (Kuschel and May 1990) and Spilopyrinae (Reid 2000). In 1997, Suzuki dissected living individuals of *Aulacoscelis* sp. (Aulacoscelidinae) from Panama for additional data on the internal reproductive systems of both sexes and provided evidence that the subfamily Aulacoscelidinae is a sister group of the subfamily Orosodacninae (Suzuki and Windsor 1999). Suzuki also examined several genera of Megalopodinae and Megascelinae (=Megascelidinae) from Panama (Suzuki 2003). Suzuki and Windsor (1999) proposed a fully revised higher phylogenetic classification system of the Chrysomelidae which is composed of nine subfamilies and 20 tribes as follows: Orsodacninae (Orsodacnini + Aulacoscelini) + Megalopodinae (Zeugophorini + Palophagini + Megalopodini) + Sagrinae (Sagrini + Donaciini + Criocerini) + Synetinae (Synetini) + Chrysomelinae (Chrysomelini) + Galerucinae (Galerucini + Alticini) + Clytrinae [(Clytrini + Cryptocephalini) + (Chlamisini + Lamprosomatini) + Cassidinae (Hispini + Cassidini) + Eumolpinae (Megascelinini + Eumolpini)].

The higher classification system which we adopted in the “New Catalog” may be behind the times, but it seems very useful as a “general reference system” because it has been widely accepted by most of the Japanese coleopterists since Chûjô and Kimoto (1961). Suzuki’s system has not been still widely accepted in Japan.

We treat here 648 species (as of in August 2023) of Japanese Chrysomelidae belonging to 172 genera in 17 subfamilies (Table 2). The number of species is considerably higher for the following five subfamilies: Alticinae (55 genera/235 species), Galerucinae (39/103), Eumolpinae (21/64), Chrysomelinae (17/60), and Cryptocephalinae (5/46). The total number of species (508) of these five subfamilies amount to about 80% of the whole Japanese fauna. The ratio of 275 indigenous species is about 42% of the whole.

**Table 2.** The number of genus and species reviewed in our “New Catalog” of the Chrysomelidae from Japan.

Subfamily	Genus	Species	Native Species (%)		Invasive Species
Megalopodinae	1	1	1	(100.0)	
Zeugophorinae	1	10	8	(80.0)	
Orsodacninae	1	1	1	(100.0)	
Synetinae	1	3	2	(66.7)	
Sagrinae	1	1	–		1
Donaciinae	3	24	8	(33.3)	
Criocerinae	4	32	6	(18.8)	4
Chrysomelinae	17	60	22	(36.7)	2
Galerucinae	39	103	50	(48.5)	1
Alticinae	55	235	117	(49.8)	2
Clytrinae	5	10	3	(30.0)	
Cryptocephalinae	5	46	13	(28.3)	
Chlamisinae	1	9	4	(44.4)	
Lamprosomatinae	2	6	4	(66.7)	
Hispinae	9	15	4	(26.7)	3
Cassidinae	6	28	4	(14.3)	2
Eumolpinae	21	64	28	(43.8)	
Total	172	648	275	(42.4)	15

The following 11 species belonging to five subfamilies are endemic to very small islands besides the four major islands (Hokkaido, Honshu, Shikoku, and Kyushu) and Okinawajima Island of the Nansei Islands: Criocerinae – Liliocelis (Lilioceris) ruficollis (Baly, 1865) (Tsushimajima Island), L. (L.) scapularis (Baly, 1859) (Tsushimajima Island); Galerucinae – *Monolepta shirozui* Kimoto, 1996 (Tsushimajima Island); Alticinae – *Parazipanginia okiana* Ohno, 1965 (Oki Islands), *Pseudoliprus komiyai* Ohno, 1966 (Koshikijima Islands); Cryptocephalinae – *Cryptocephalus ejimai* Takizawa, 1990 (Danjo-guntô Islands); Eumolpinae – *Basilepta amamiensis* Chûjô, 1957 (Amami-Oshima Island), *B. borodinensis* Kimoto, 1979 (North and South Daitôjima Islands), *Colaspoides kasaharai* Komiya, 1991 (Yonagunijima Island), *C. suginoi* Komiya, 1988 (Tokunoshima Island), *Phytorus lineatus* Weise, 1913 (Iôtô Island in the Ogasawara Islands).

The following three species belonging to two subfamilies should be deleted from the Japanese fauna because they were accidentally recorded: Chrysomelinae – *Agrosteomela indica* (Hope, 1831) (Nagano, Honshu), *Timarcha punctella schrammi* Kocher, 1957 (Fukushima, Honshu); Cryptocephalinae – Cryptocephalus (Cryptocephalus) trifasciatus Fabricius, 1787 (Kyushu).

The following 15 species belonging to seven subfamilies were introduced to Japan after the beginning of the Meiji era (1868–1930) and indisputably naturalized (included inside parentheses for each is the year of introduction into Japan along with the presumed country of origin): Sagrinae – Sagra (Sagra) femorata (Drury, 1773) (2011/Thailand); Criocerinae – Lema (Lema) diversipes Pic, 1921 (2018/Taiwan), L. (L.) lacertosa Lacordaire, 1845 (2016/Taiwan), L. (L.) trivittata Say, 1824 (?/?), L. (Microlema) pectoralis Baly, 1865 (?/Taiwan); Chrysomelinae – Plagiostern a formosana (Bates, 1866) (2017/Taiwan), *Trachymela sloanei* Blackburn, 1897 (2008/Australia); Galerucinae – *Ophraella communa* LeSage, 1986 (1996/North America); Alticinae – *Disonycha poly­tula* Horn, 1889 (2023/North America?), *Epitrix hirtipennis* (Melsheimer, 1847) (2012/North America); Hispinae – *Brontispa longissima* Gestro, 1885 (?/?), ?*Platypria echidna* (Guérin-Méneville, 1840) (?/?), *P. melli* Uhmann, 1955 (?/?); Cassidinae – *Laccoptera nepalensis* Bohemann, 1855 (?/?), Cassida (Taiwania) obtusata Bohemann, 1854 (?/?). About half of these 15 species were apparently introduced into Japan before 2000. Recently the number of invasive species in Japan is increasing rapidly; that is, at least seven species have been introduced to Japan since 2000. Among them, it should be especially noted that *Sagra femorata* was introduced from Thailand in 2011 (Akita et al. 2011) and quickly settled in Japan as the single member of the 17^th^ chrysomelid subfamily in Japan. Many Japanese coleopterists think that some individuals of this *Sagra* were under captive breeding in pet shops which sold small animals including insects imported from Thailand. This species then escaped and settled quickly because the wild kudzu vine, *Pueraria lobata* (Willd.) Ohwi (Fabaceae), one of their preferable host plants, is common in parts of Japan.

### ﻿Studies of local fauna in the family Chrysomelidae from Japan

The local chrysomelid fauna in Japan has been well-studied by a number of professional and non-professional workers who are interested in the Chrysomelidae, although the depth varies in each region. There are probably more than 200 local associations for study of insects in Japan; most were established for lovers of specific insect taxa and/or for particular geographical regions since the beginning of the 20^th^ century and most publish a journal. Clarification of the local insect fauna is largely due to these local associations.

**Figure 1.**
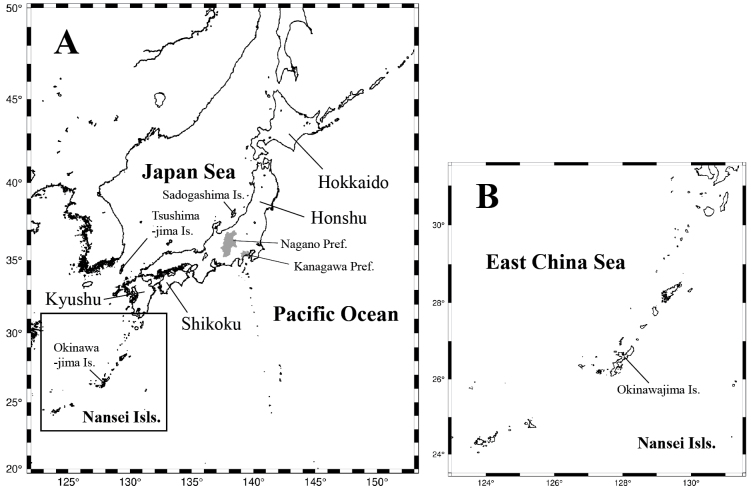
**A** map of Japan. Names of the 10 representative regions selected for comparison are indicated; the four major islands of Hokkaido, Honshu, Shikoku and Kyushu, the Nansei Islands, the three zoogeographicaly important islands of Sadogashima, Tsushimajima, and Okinawajima, and Kanagawa and Nagano prefectures of central Honshu **B** position of Okinawajima Island within the Nansei Islands.

Based on a large amount of information accumulated so far, we examined the local fauna of each of the four major islands, the Nansei Islands, three representative islands (Sadogashima and Tsushimajima Islands in the Sea of Japan and Okinawajima Island in the Nansei Islands), and two representative prefectures of central Honshu (Kanagawa and Nagano Prefectures). Table 3 shows the details of the chrysomelid fauna in each of 10 regions (see Fig. 1). The details of the data in two prefectures were cited from Takizawa (2018a) for Kanagawa Prefecture and Takizawa (2018b) for Nagano Prefecture. The chrysomelid faunas of these two prefectures have been the most exhaustively surveyed among the 47 administrative divisions of Japan.

**Table 3.** Comparison of the chrysomelid fauna among 10 regions in Japan.

Region	Area km^2^ (%)	Subfamily (%)	Genus (%)	Species (%)
Whole of Japan	378,000 (100.0)	17 (100.0)	172 (100.0)	648 (100.0)
1 Hokkaido	78,000 (20.6)	13 (76.5)	74 (43.0)	205 (30.9)
2 Honshu	227,900 (60.3)	17 (100.0)	137 (79.7)	336 (51.9)
3 Shikoku	18,800 (5.0)	16 (94.1)	107 (62.2)	257 (39.7)
4 Kyushu	36,800 (9.7)	16 (94.1)	124 (72.1)	324 (50.0)
5 Nansei Isls.	3,800 (1.0)	13 (76.5)	100 (58.1)	239 (36.9)
6 Sadogashima I.	855 (0.2)	13 (76.5)	50 (29.1)	73 (11.3)
7 Tsushimajima I.	700 (0.2)	13 (76.5)	67 (39.0)	141 (21.8)
8 Okinawajima I.	1,200 (0.3)	13 (76.5)	66 (38.4)	94 (14.5)
9 Kanagawa Pref.	2,400 (0.6)	16 (94.1)	118 (68.6)	307 (47.4)
10 Nagano Pref.	13,500 (3.6)	16 (94.1)	106 (61.6)	289 (44.6)

Tables 4, 5 show the details of the number of genera (Table 4) and species (Table 5) in the 10 regions of Map A.

**Table 4.** Comparison of the number of genera among 10 regions in Japan. Abbreviations—Subf.: subfamily, MEG: Megalopodinae, ZEU: Zeugophorinae, ORS: Orsodacninae, SYN: Synetinae, SAG: Sagrinae, DON: Donaciinae, CRI: Criocerinae, CHR: Chrysomelinae, GAL: Galerucinae, ALT: Alticinae, CLY: Clytrinae, CRY: Cryptocephalinae, CHL: Chlamisinae, LAM: Lamprosomatinae, HIS: Hispinae, CAS: Cassidinae, EUM: Eumolpinae, Jpn: whole of Japan, Hok: Hokkaido, Hon: Honshu, Shi: Shikoku, Kyu: Kyushu, Nan: Nansei Islands, Sad: Sadogashima Island, Tsu: Tshushimajima Island, Oki: Okinawajima Island (Nansei Islands), Kan: Kanagawa Prefecture (Honshu), Nag: Nagano Prefecture (Honshu).

Subf.	Jpn	Hok	Hon	Shi	Kyu	Nan	Sad	Tsu	Oki	Kan	Nag
MEG	1	–	1	1	1	–	–	–	–	1	1
ZEU	1	1	1	1	1	1	–	1	1	1	1
ORS	1	–	1	1	1	–	–	–	–	1	1
SYN	1	1	1	1	1	–	–	1	–	1	1
SAG	1	–	1	–	–	–	–	–	–	–	–
DON	3	3	3	2	3	1	2	–	–	3	2
CRI	4	4	4	3	4	3	3	4	3	4	4
CHR	17	10	17	10	12	8	7	5	5	11	12
GAL	39	18	31	23	29	20	12	13	10	28	27
ALT	55	23	43	34	38	31	14	23	24	38	29
CLY	5	1	4	4	4	3	1	2	1	3	3
CRY	5	2	4	4	4	4	2	2	3	4	3
CHL	1	–	1	1	1	1	1	1	1	1	1
LAM	2	1	2	2	2	1	1	1	1	2	2
HIS	9	1	4	3	4	6	1	1	2	3	3
CAS	6	4	4	3	4	4	1	3	4	4	4
EUM	21	5	15	14	15	17	5	10	11	13	12
	172	74	137	107	124	100	50	67	66	118	106
	100.0	43.0	79.7	62.2	72.1	58.1	29.1	39.0	38.4	68.6	61.6

**Table 5.** Comparison of the number of species among 10 regions in Japan. Abbreviations—see Table 4.

Subf.	Jpn	Hok	Hon	Shi	Kyu	Nan	Sad	Tsu	Oki	Kan	Nag
MEG	1	–	1	1	1	–	–	–	–	1	1
ZEU	10	4	6	4	4	2	–	1	1	5	5
ORS	1	–	1	1	1	–	–	–	–	1	1
SYN	3	2	3	1	1	–	–	1	–	1	2
SAG	1	–	1	–	–	–	–	–	–	–	–
DON	24	6	20	3	7	1	4	–	–	5	11
CRI	32	10	25	17	23	14	7	14	3	19	17
CHR	60	32	43	17	19	9	10	6	5	21	29
GAL	103	33	68	43	52	42	14	20	16	55	52
ALT	235	76	154	94	118	92	22	49	40	113	102
CLY	10	1	7	6	7	4	1	3	2	5	4
CRY	46	18	32	18	22	7	3	9	4	23	22
CHL	9	–	7	4	7	3	1	3	2	7	3
LAM	6	2	3	3	3	4	1	2	2	3	3
HIS	15	2	8	6	8	6	1	3	2	5	5
CAS	28	12	23	11	13	10	4	10	9	14	15
EUM	64	7	34	28	38	45	5	20	18	29	17
	648	205	336	257	324	239	73	141	94	307	289
	100.0	31.6	51.9	39.7	50.0	36.9	11.3	21.8	14.5	47.4	44.6

Hokkaido, in the southernmost area of the Subarctic Zone, has relatively limited vegetation. As a result, the number of the chrysomelid species is confined to about 32% of the entire Japanese fauna. In contrast, the remaining three more southern major islands (Honshu, Shikoku, and Kyushu) along with the Nansei Islands belong to the Temperate and Subtropical Zones, where vegetation is abundant and diverse. The total number of species for these three major islands is about 40–50% of the total fauna. We note that both total number of known species in the two local regions Kanagawa and Nagano Prefectures, reach approximately 45% of the whole Japanese fauna and reaches more than 85% for all of Honshu.

Based on the comparison of these data we can see many remarkable facts and tendencies of the chrysomelid faunas of the 10 regions, as follows:

(1) Hokkaido belongs to the Subarctic Zone and occupies about 20% of the area of Japan. Its flora is relatively non-diverse which is reflected in the low levels of genera and species (43% and 32%, respectively) of the entire fauna.

(2) The three major islands of Honshu, Shikoku, and Kyushu belong to the Temperate Zone. Honshu occupies about 60% of the area of Japan and has a long mountain chain which runs for about 1,500 km from the northeast to southwest and provides complex, diverse natural environments; about 80% of the genera and 52% of the species of Japan are distributed on Honshu alone.

(3) Shikoku occupies only 5% of the area of Japan but has 62% of the genera and 40% of the species.

(4) Kyushu occupies less than 10% of the area in Japan but has 72% of the genera and 50% of the species.

(5) The Nansei Islands belong to the Subtropical Zone and occupy only 1% of the area in Japan but contains 58% of the genera and 37% of the species.

(6) Three zoogeographically important islands (Sadogashima, Tsushimajima, and Okinawajima) occupy a mere 0.2–0.3% of Japan but have 30–39% of the genera and 11–22% of the species, respectively.

(7) Two limited regions of central Honshu (Kanagawa and Nagano Prefectures) occupy 0.6 and 3.6%, respectively of Japan but 69% and 62% of the genera and 47% and 45% of the whole fauna. Both Kanagawa and Nagano Prefectures have been well-studied and if unidentified species were added these figures would be close to those of all of Honshu.

### ﻿A summarized history of systematic studies of Chrysomelidae from Japan

Since the beginning of the Meiji era (1868–1911) scientific practices from western countries have been introduced into Japan. In Japan, entomologists began to study economically important pests including many chrysomelid species, such as *Phaedon brassicae* Baly, 1874 (Chrysomelinae; HP (host plant): Brassicaceae); *Aulacophora indica* (Gmelin, 1790) (Galerucinae; HP: Cucurbitaceae); *Phyllotreta striolata* (Fabricius, 1803) (Alticinae; HP: Brassicaceae); *Psylliodes* spp. (Alticinae; HP: Solanaceae); *Basilepta pallidula* (Baly, 1874) (Eumolpinae; HP: Cupressaceae) and some others, in the departments of natural science, agriculture and forestry in universities and various local institutes. Some have been used as model species in various fields of zoology. The fundamental framework of systems of classification of the Chrysomelidae in Japan was not well established until the 1880’s by Baly, Jacoby, Motschulsky, Weise and some other European coleopterists. At that time 250 species belonging to 16 subfamilies were already known.

**Figure 2.**
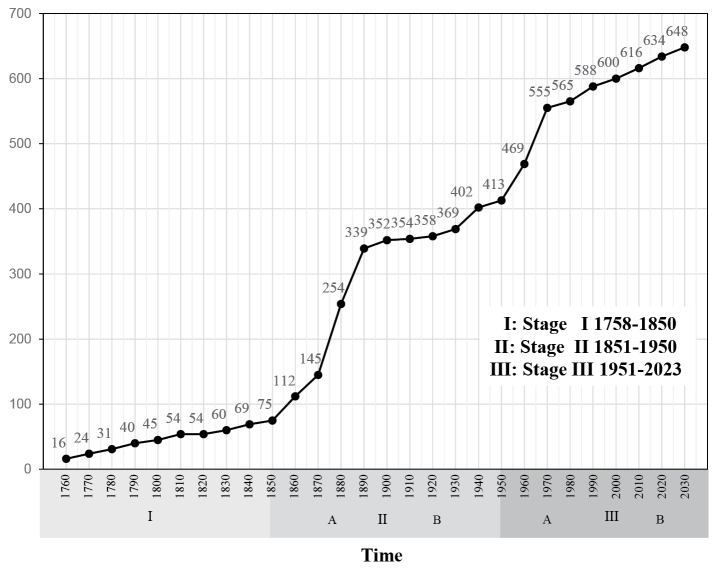
Cumulative number of the Japanese chrysomelid species described.

We examined the transition of the number of species described during every 10 years period from 1750 to the present. Figure 2 shows the cumulative curve of the total number of the described Japanese species over those periods.

In this paper, we treat the history of studies of the Japanese fauna of Chrysomelidae, which we divide into three stages: Stage I (1751–1850), Stage II (1851–1950), and Stage III (1951 to the present). The curve of cumulative number of species described in Fig. 2 clearly demonstrates some remarkable tendencies; that is, it gently increases in Stage I, rapidly increases in the first half of Stage II but subsequently there is a noticeable flat state apparent in the second half and rapidly increases again in the first half of Stage III, then smoothly increases in its latter half. The rapid increase in the first half of Stage II indicates the contributions of European coleopterists like Baly, Jacoby, Motschulsky, Weise, and others.

By the 1910’s, about 350 species belonging to 16 subfamilies were known. The flat situation in the second half of Stage II reflects the effects of World Wars I and II. The second obvious increase in Stage III can be regarded as a significant development in science and technology and interchange among scientists from various countries. The same tendencies are obvious when we examined four subfamilies that contain relatively many species: Alticinae (55 genera/235 species), Galerucinae (39/103), Eumolpinae (21/64), and Chrysomelinae (17/60).

Based on the conventionally and generally accepted concepts of the genus and species, we assume knowledge of Japanese chrysomelid fauna had almost reached to 95% at the generic level and to 80% at the specific one by the latter half of this century.

Chûjô (1953–1954) published papers commemorating the history of system­atic study in Japanese entomology at the beginning of Stage III. He discussed and proposed a phylogenetic system of classification of the Chrysomel­idae based on the comparative morphology of several phylogenetic characters (male external genitalia, hind wing venation, etc.). For details of a phylogenetic system of higher classification taxa, especially at the subfamily level for Chrysomelidae, refer to historical reviews of Suzuki (1988, 1994a, 1994b, 1996, 2003) and Suzuki and Windsor (1999).

The 10 coleopterists who described the most Japanese taxa of Chrysomelidae are listed in Table 6 as follows (in parenthesis are the number of described species, their percentage compared to the total number of Japanese taxa, and the publication period): Baly [109 (16.8%), 1859–1886]; Jacoby [68 (10.5%), 1884–1896]; Chûjô [66 (10.2%), 1935–1967]; Kimoto [48 (7.4%), 1957–1995]; Takizawa [35 (5.4%), 1970–2021]; Motschulsky [35 (5.4%), 1854–1866]; Ohno [32 (4.9%), 1958–1968]; Nakane [24 (3.7%), 1954–1985]; Linnaeus [22 (3.4%), 1758–1768]; Weise [13 (2.2%), 1878–1913]. These 10 workers described a total of 452 species (about 70% of the currently known 648 species including 22 Linnaean species).

**Table 6.** Ten representative entomologists who studied the Japanese fauna of Chrysomelidae based on the number of the species they described. Abbreviations of subfamily names—see Table 4.

Subf.	Baly	Jacoby	Chûjô	Kimoto	Motschulsky	Takizawa	Ohno	Nakane	Linnaeus	Weise
MEG	1	–	–	–	–	–	–	–	–	–
ZEU	1	3	4	–	–	–	1	–	–	–
ORS	–	–	1	–	–	–	–	–	–	–
SYN	1	–	–	1	–	–	–	1	–	–
SAG	–	–	–	–	–	–	–	–	–	–
DON	–	3	1	2	–	–	–	2	1	1
CRI	12	3	1	–	1	–	1	–	2	–
CHR	4	2	2	4	5	13	–	2	5	2
GAL	15	14	11	15	11	–	1	5	3	4
ALT	38	30	21	19	10	17	26	11	2	3
CLY	1	1	3	–	–	–	–	–	1	1
CRY	12	4	4	1	–	4	–	–	2	1
CHL	3	2	2	–	–	–	1	–	–	–
LAM	2	1	2	1	–	–	–	–	–	–
HIS	2	–	2	2	1	1	–	–	–	–
CAS	2	–	2	–	2	–	–	–	5	–
EUM	15	5	10	3	5	–	2	3	1	1
	109	68	66	48	35	35	32	24	22	13

Therefore, we are confronted with many problems of classification of the Japanese Chrysomelidae. To establish a more reliable system of classification we should carefully compare the differences among various hypotheses obtained from different sources; for example, the criteria for determining the taxonomic rank of a given taxon may differ among classifications. How should we determine a taxonomic rank of a given taxon? This was always a source of distress for author Suzuki (since his graduate student days) in considering the ideal phylogenetic classification system for Chrysomelidae, which could be inferred from comparison of different proposed systems and data types.

Table 7 shows six representative works, including illustrated reference books and comprehensive systematic catalogs of the Japanese Chrysomelidae published in the last 65 years. From this we can see the entire status of the Japanese chrysomelid fauna at a particular point in time. The publications by Chûjô and Kimoto (1961), Ohno (1971), and Kimoto (1994) treated all the known Japanese Chrysomelidae. It was unavoidable that Nakane (1963) and Kimoto (1984) were limited in their treatment of genera and species because both these are general illustrated publications. Table 7 well reflects the essence of the history of study of the Japanese chrysomelid fauna.

**Table 7.** The number of genus and species treated in 6 representative catalogs by Japanese workers. A: total number of genera and species treated; B: percent (%) of total number of genera and species treated against those in our “New Catalog”. Abbreviations of subfamily names—see Table 4.

Subf.	Chujo and Kimoto (1961)		Nakane (1963)		Ohno (1971)		Kimoto (1984)		Kimoto (1994)		Takizawa and Suzuki (in press)	
	Genera	Species	Genera	Species	Genera	Species	Genera	Species	Genera	Species	Genera	Species
MEG	1	1	1	1	1	1	1	1	1	1	1	1
ZEU	1	8	1	5	1	10	1	9	1	9	1	10
ORS	1	1	1	1	1	1	1	1	1	1	1	1
SYN	1	1	1	1	1	2	1	2	1	2	1	3
SAG	–	–	–	–	–	–	–	–	–	–	1	1
DON	3	12	3	13	3	17	3	17	3	20	3	24
CRI	4	27	4	23	4	30	4	27	4	28	4	32
CHR	16	32	14	32	17	49	12	41	15	46	17	60
GAL	37	75	32	63	37	98	38	94	39	97	39	103
ALT	47	141	38	90	49	193	49	175	49	193	55	235
CLY	3	10	3	9	3	10	4	9	4	9	5	10
CRY	4	38	4	26	4	42	4	37	5	32	5	46
CHL	1	8	1	5	1	9	1	9	1	9	1	9
LAM	2	5	2	3	2	7	2	7	2	6	2	6
HIS	6	11	3	7	7	13	6	11	9	14	9	15
CAS	6	28	3	16	7	30	6	23	6	25	6	28
EUM	20	42	18	35	20	50	17	44	21	61	21	64
A	153	440	129	330	158	562	152	507	162	553	172	648
B	90.0	67.9	75.0	50.9	91.9	86.7	88.4	78.2	94.2	85.3	100	100

## ﻿Additional information

### Use of AI

No use of AI was reported.

### Author ORCIDs

Haruo Takizawa https://orcid.org/0009-0000-6992-4998

### Data availability

All of the data that support the findings of this study are available in the main text.
